# The Prone Position during Operations upon the Jaws

**Published:** 1883-10

**Authors:** L. McLane Tiffany

**Affiliations:** Professor of Surgery, University of Maryland.


AMERICAN JOURNAL

OF

DENTAL SCIENCE.

VOL. XVII. THIRD SERIES—OCTOBER, 1883. No. 6.

ARTICLE I.

### THE PRONE POSITION DURING OPERATIONS UPON THE JAWS.

BY L. MCLANE TIFFANY, M. D.

Professor of Surgery, University of Maryland.

GENTLEMEN: I have the honor to bring this patient before you to-day, to show not only the small amount of deformity which may be present after the removal of a large portion of the upper jaw, but also, and this is even of more importance, to speak of a method of operating whereby greater facility of manipulation is secured to the surgeon. Until the discovery of anæsthesia, operations upon the upper jaw, or indeed either jaw, were undertaken while the patient was in a sitting posture, usually leaning somewhat forwards, in order to facilitate the escape of blood outwards through the lips.

The danger of retaining a patient in the erect position during the induction and continuance of profound anæsthesia is well known, however, so that the patient's desire for chloroform or ether, aud the operator's desire to afford a free exit for blood from the mouth, other than by the windpipe, has caused many expedients to be practiced; expedients, by the way, which it may be worth while to mention. The sitting posture has been referred to, which may be preceded or not by a full dose of opium; inversion more or less complete, usually undertaken hurridly to avoid asphyxia from unexpected escape ot blood into the trachea, as has occurred to me once;* Rose's position, the head hanging over the edge of the table so as to bring the vault of the pharynx downwards, thus transforming it into a cup from which blood may be sponged as required; in the supine position, tracheotomy combined with occlusion of the trachea above the artificial opening, by an inflatable bag, by wrappnig the tracheotomy tube that it shall fill the windpipe, or by stuffing the pharynx with sponge; by introducing a tube into the air passage through the mouth and partially filling the pharynx with sponge, etc. All these and many other expedients have probably been put in practice with beneficial results, doubtless, for it can be said without fear of contradiction that no method of operating but is sometimes indicated and sometimes contraindicted. In general it is accepted that the more simply an operation is conducted the better, and that a wound in the mouth should not be complicated by a wound in the trachea unless absolutely necessary.

In a paper read before this Faculty some time since,* I called attention to the prone position as offering certain advantages, the patient's head being somewhat raised, facing a window. At that time I had had recourse to it upon two occasions when excising portions of the upper jaw; since the date mentioned I have had recourse to it several times, and see no reason to modify the favorable opinion then expressed.

The advantage to be gained when operating upon the jaws by semi-narcotizing the patient with opium or one of its alkaloids is very great, since sensation is abolished before profound unconsciousness is induced by ether or

*Trans. Med. and Chi. Fac. of Md., 1876, p. 181.

*Trans. Med. and Chi. Fac., 1880, p. 207.

chloroform. In several instances I have been told by patients that they had suffered no pain during operative measures, although they had obeyed directions in a loud tone of voice during the progress of the operation. Confirmative evidence to the above is seen by the quietude of patients during operation, when treated as suggested.

The opiate should be given a certain time before the anæsthetic is inhaled, morphine hypodermically (not less than than thirty minutes); a full dose is required, and that the patient have no idiosyncrasy, it is wise to give a tentative dose a day or two previously, especially in children.

The position to which I have referred as being advantageous is as follows: The patient having received a full dose of morphine, hypodermically, and subsequently anæsthetized, is turned face down, the upper portion of the thorax extends beyond the table, being supported by an assistant at each shoulder—respiration is thus less labored. A third assistant supports the head, standing behind the shoulder carrier, so as to be out of the operator's way. The face being raised towards a window, light is obtained, while blood from incisions, etc., runs out of the mouth into some vessel placed conveniently. The operation upon the patient, now shown to the Faculty, was the most extensive in the position referred to that I have done. The portion of upper jaw removed extended from the left lateral incisor to the last right molar; both nares were opened, yet bleeding caused no anxiety, for it flowed from, not towards, the larynx and escaped externally.

I venture to think that the prone position may permit the removal of an upper jaw, or a pharyngeal tumor, without a preliminary tracheotomy.

The case presented is as follows: A—— B——, female, menstruation not yet present, æt. 15 years, has noticed swelling of right upper jaw during past two years, no cause known. Latterly increase in size has been marked, no pains of discomfort experienced. Examination shows a firm, elastic tumor of right upper jaw, entirely surrounded by bone, apparently about one inch and a quarter in diameter, encroaching on the intermaxillary suture. The right lateral incisor had erupted very late, and was of imperfect development. Presumptively the tooth and tumor were connected.

Three weeks later A—— B—— was again seen; the tumor had increased decidedly, absorption of right palate process had taken place, and fluctation at one part of growth was apparent. After a few days preparatory treatment, the tumor, together with adjacent bone, was removed, strong bone forceps being used as being more rapid in execution than a saw. The pliers were of different shapes, the blades being at an angle with the handles. The upper lip was split in the middle line to facilitate bone removal. The after treatment consisted of great cleanliness by means of warm carbolized water. Secondary hemorrhage from both nostrils occurred on the fourth day, requiring plugging. Afterwards all went well, and the patient returned home in three weeks. The tumor was partly solid, partly cystic, and was connected with the fang of the immature lateral incisor Microscopic examination showed it to be a spindle-called sarcoma.

